# Palliative Care Needs in Advanced Non-Malignant Chronic Conditions: A Qualitative Study of Greek Patients’ and Caregivers’ Perspectives

**DOI:** 10.3390/healthcare14040479

**Published:** 2026-02-13

**Authors:** Chrysovalantis Karagkounis, Christina Papachristou, Evgenia Minasidou, Thalia Bellali

**Affiliations:** 1 Nursing Department, International Hellenic University, Sindos, 57400 Thessaloniki, Greece; kchrysov@gmail.com (C.K.); minasidu@ihu.gr (E.M.); 2 School of Psychology, Faculty of Philosophy, Aristotle University of Thessaloniki, University Campus, 54124 Thessaloniki, Greece; cpapachr@psy.auth.gr; 3 Department of Health Sciences, European University Cyprus, Engomi, Nicosia 1516, Cyprus

**Keywords:** caregivers, chronic non-malignant diseases, holistic care, patients, palliative care, qualitative study, support needs

## Abstract

**Highlights:**

**What are the main findings?**

- Patients with advanced non-malignant chronic conditions and their caregivers experience substantial unmet needs across daily physical care, psychosomatic burden, social isolation, and navigation of the healthcare system.
- Family support and spiritual practices function as key resilience mechanisms, yet cannot compensate for systemic gaps such as a lack of home-based physiotherapy/nursing, caregiver training, and structured psychological support.

**What are the implications of the main findings?**

- The findings highlight the urgent need to develop community-based, integrated palliative care models that provide home-based clinical services, caregiver respite, and psychosocial support tailored to non-malignant chronic conditions.
- The results support the future development and validation of a multidimensional needs-assessment tool to guide referral pathways, strengthen caregiver preparedness, and inform policy initiatives for equitable palliative care access.

**Abstract:**

Background/Objectives: Palliative care (PC) has traditionally focused on patients with cancer and their families. However, individuals living with advanced non-malignant chronic diseases and their caregivers face comparable challenges that significantly affect their quality of life. This study aimed to explore the PC needs of patients with advanced non-malignant chronic conditions through the lived experiences of both patients and their informal caregivers. Methods: Semi-structured interviews were conducted with eight patients and nine caregivers recruited via the Municipality of Katerini “Help at Home” program (Jan–Mar 2025). Interviews were audio-recorded, transcribed verbatim (in Greek), and analyzed inductively using reflexive thematic analysis. Ethical approval was obtained from the International Hellenic University (Ref. No. 18/22.12.2022), and official consent was gained from the Municipality of Katerini (Approval Ref. No. 7803-/30/01/2025). Results: Five themes emerged: (1) basic daily care and physical support; (2) psychosomatic and emotional impact; (3) social withdrawal and role change; (4) support systems and coping resources; and (5) experience with the healthcare system and organized care. Participants highlighted urgent needs for home-based physiotherapy/nursing, caregiver respite, and psychological support. Coping and resilience-related resources—expressed through family support, familiarity of the home environment, and spirituality—were described as essential mechanisms that helped dyads sustain home care and shaped how needs were experienced across multiple domains, particularly amid service gaps. Conclusions: These findings document complex, interlinked needs among patients with advanced non-malignant chronic conditions and their caregivers and support the development of community-based, integrated PC services. Larger, multicenter studies and the development/validation of a needs-assessment tool are recommended.

## 1. Introduction

Palliative care (PC) is a holistic approach designed to improve the quality of life for patients and their families facing health challenges associated with life-threatening illnesses. It focuses on preventing and alleviating suffering by addressing physical, psychological, social, and spiritual needs. [1]. While initially developed for patients with terminal cancer, the principles and applications of PC have expanded to encompass a broader range of chronic and life-threatening conditions, including chronic obstructive pulmonary disease (COPD), end-stage renal disease (ESRD), heart failure, and other progressive illnesses [2,3,4]. Such populations are commonly described in the literature as patients with advanced non-malignant chronic conditions [5,6,7].

Globally, the need for PC has increased significantly: an estimated 73.5 million people experience severe health-related suffering that could be alleviated with adequate care [8]. Despite this need, coverage remains limited, with only about 14% of those who need PC receiving such services [9]. By 2040, projected increases in deaths from chronic illnesses—particularly among older age groups—further underscore the need for integrated, age-sensitive, and diagnosis-inclusive PC services [10]. Advanced non-malignant chronic conditions represent a substantial proportion of the total population in need of PC worldwide [1], yet these patients remain underserved compared with cancer populations, despite comparable multidimensional symptom burden [3,6,7,11,12,13,14]. Needs are disproportionately concentrated among older adults due to population aging, multimorbidity, and functional decline [1,6], while younger people with advanced non-malignant disease often face prolonged and unpredictable trajectories and substantial symptom burden, but are still less likely to be referred to specialist PC services [5,6].

According to comparative evidence reported by Alnajar et al. [11], PC services remain largely structured around cancer populations and oncology-driven referral pathways, and therefore often fail to adequately address the heterogeneous and prolonged needs of patients with advanced non-malignant chronic conditions. Patients with these conditions experience similar burdens of physical, psychological, social, and spiritual symptoms as patients with cancer, but are significantly less likely to access PC services [11,12,13,14]. They are also at higher risk of intensive care hospitalizations and receiving life-sustaining treatments compared to patients with cancer [15,16,17]. This discrepancy is partly driven by limited awareness and understanding of PC needs for advanced non-malignant chronic conditions among healthcare providers and systems [3].

Despite recognition of these disparities, much of the literature has focused primarily on physical aspects of care, limiting a comprehensive understanding of multidimensional needs in everyday life—particularly in community and home settings [18,19,20]. Consequently, tailored PC models that holistically address physical, psychological, social, and spiritual needs are needed for patients with advanced non-malignant chronic conditions and their families [17,21].

Caregivers play a pivotal role in PC, particularly for patients who require long-term home support. Caregivers monitor treatment, manage symptoms, and provide emotional, financial, and spiritual support [22]. However, caregiving responsibilities can impose significant physical, psychological, and social burdens, often exacerbated by insufficient preparation and limited access to resources [23]. Studies have highlighted that caregivers frequently feel unprepared to fulfill their roles, lacking adequate knowledge about the disease, caregiving techniques, and available resources [23,24,25]. This limited preparedness can compromise the quality and sustainability of home-based care and negatively affect caregivers’ well-being [24]. Moreover, in community and home-based care, informal caregivers often function as co-providers of PC; insufficient preparedness may directly affect symptom management, continuity of care, and potentially avoidable healthcare utilization, while also undermining caregivers’ own well-being [24,26].

PC in Greece remains underdeveloped compared to other high-income European Union countries. The Ministry of Health’s Palliative Care Feasibility Study for Greece (2019), funded by the Stavros Niarchos Foundation, suggests that service availability remains limited relative to estimated need [27]. Only three specialized PC programs operate nationwide, serving approximately 600 patients annually, while the estimated need far exceeds available resources (approximately 120,000 to 135,000 patients and families per year) [27]. The majority of patients requiring PC in Greece are patients with advanced non-malignant chronic conditions (63%), and the lack of sufficiently resourced home care teams and inpatient facilities limits the capacity to support home-based care and death, despite patient preferences [27].

Beyond documenting unmet needs, it is also essential—within a holistic PC perspective—to consider coping resources (e.g., family, faith, and community support) that may shape how needs are perceived and managed in everyday community and home care [1,20]. Accordingly, this study explores multidimensional PC needs and addresses coping resources (including resilience-related resources) as part of patients’ and caregivers’ lived experiences in the community.

Overall, while international evidence documents inequities for non-malignant populations, in Greece, qualitative evidence remains very limited—particularly in-depth research that simultaneously captures both patients’ and informal caregivers’ perspectives on PC needs in community/home contexts among people living with advanced non-malignant chronic conditions. To bridge this gap, it is essential to prioritize the development of community-based PC services tailored to the needs of patients with advanced non-malignant chronic conditions and their caregivers, emphasizing early integration, improved symptom management, quality of life, and reduced healthcare utilization [28,29,30]. Given the substantial unmet needs of patients with advanced non-malignant chronic conditions and the significant burden on their caregivers, this study aims to explore patients’ PC needs qualitatively. By examining the experiences and challenges of both patients and caregivers, this study seeks to:

1. Identify the specific physical, psychological, social, and spiritual needs of patients with advanced non-malignant chronic conditions.
2. Explore caregivers’ preparedness and support requirements in their role as providers of care, as these relate to the identification and management of patients’ PC needs.
3. Inform the development of tailored PC models that address the unique characteristics of patients with advanced non-malignant chronic conditions and their caregivers.

Furthermore, by investigating their needs, we hope to generate valuable insights that can inform the development of a new tool to assess the needs of patients with advanced non-malignant chronic conditions in PC services. This tool will be accessible and usable by all stakeholders, including patients, caregivers, and healthcare professionals. By addressing these objectives, this study aims to contribute to the growing body of evidence supporting equitable and holistic PC services for patients with advanced non-malignant chronic conditions and their families.

## 2. Materials and Methods

### 2.1. Participant Selection and Study Procedures

#### Sampling Strategy

A purposive sampling strategy was employed to recruit information-rich cases relevant to the study aims. Specifically, maximum variation purposive sampling was used to capture a heterogeneous range of experiences across key characteristics, including age, type of advanced non-malignant chronic condition, functional status, and caregiving context. The a priori axes of variation were: (i) diagnosis group/clinical profile (e.g., cardiopulmonary, neurological, renal/other organ failure), (ii) functional status and care intensity (e.g., ambulatory vs. mobility-limited/bedbound; need for assistance in ADLs), (iii) caregiving relationship and living arrangement (e.g., spouse/partner vs. adult child/other relative; co-residing vs. non–co-residing), and (iv) place of residence (urban vs. rural areas within the Municipality of Katerini). This strategy was selected to maximize the breadth of lived experiences relevant to the study aims and to recruit information-rich cases, consistent with qualitative sampling principles and the concept of information power.

Participants were recruited from two groups: patients with advanced non-malignant chronic conditions and their caregivers. Recruitment and eligibility criteria were designed to ensure the inclusion of participants who could provide rich, meaningful qualitative data rather than statistical representativeness. The eligibility criteria for both groups are summarized in Table 1. Patients with neurological diseases (e.g., ALS, MS) were eligible for inclusion, provided that their communication abilities were sufficiently preserved to participate in in-depth interviews. Patients with neurological or psychiatric conditions were excluded only when communication impairments prevented meaningful contribution to the interview process.

Through the Municipality of Katerini’s “Help at Home” program, sponsored by the Department of Social Policy and Public Health’s Division of Elderly Care, participants were recruited. The staff assisted in identifying and establishing the primary contact for eligible people. Prospective participants were provided with written and verbal information about the study’s goals, procedures, and requirements. A standard screening procedure was adopted to ascertain adherence to the inclusion and exclusion criteria outlined in advance. The study received ethical approval from the Research Ethics and Bioethics Committee of the International Hellenic University (Approval Ref. No. 18/22.12.2022). In addition, permission to recruit participants through the “Help at Home” program was granted by the Municipality of Katerini, Department of Social Policy and Public Health (Approval Ref. No. [7803/30.01.2025]). Both approvals were obtained before data collection commenced. Recruitment and data collection were conducted between January and March 2025.

All participants provided written informed consent before being included in the study. Data were gathered through qualitative interviews to ascertain the physical, emotional, and social aspects of PC needs. The interviews took place at the patients’ homes to accommodate individual needs and preferences. Given the possibility that a few participants might experience mobility issues, great care was taken to ensure their full participation without the added burden of travel. Being interviewed in their usual environment might have been very comforting and reassuring for participants, allowing them to relax and be more open to genuine responses. At the same time, the home atmosphere enabled participants to express themselves endearingly, as they could easily relate their expressions to their day-to-day lives and culture, adding depth and relevance to the data being collected. The study’s design maintained an accessible and inclusive spirit to obtain a sample that reflected diverse social, age, and health profiles. With this approach, the study endeavored to comprehensively capture the PC needs of patients with advanced non-malignant chronic conditions and their caregivers. Communication ability was assessed during the screening process by the research team, in collaboration with ‘Help at Home’ staff, to ensure that participants could engage with open-ended interview questions.

### 2.2. Qualitative Interviews

Qualitative, semi-structured interviews were conducted with individuals who met the eligibility criteria. Study participants comprised patients with advanced non-malignant chronic conditions and their primary informal caregivers admitted to the “Help at Home” program of the Municipality of Katerini. The recruitment and enrollment process was conducted between January and March 2025; of the 15 patients and 16 caregivers approached, eight patients and nine caregivers provided consent to participate. Reasons provided by people who refused to be interviewed were poor health prognosis (physical/emotional) (5), lack of interest or time (4), or inability to agree on a convenient time due to logistical or family constraints (5). Purposive sampling ensured variation in participants’ caregiving experience, types of chronic illness, and levels of support needed. The objective was to encompass multiple perspectives, including older and younger populations, men and women, and those from urban and rural areas of Katerini. The recruitment process was completed when thematic saturation was observed, as no new codes or themes emerged in the last two interviews. Saturation was assessed at the code and candidate theme levels using a practical criterion. After each interview, the interviewer produced a brief analytic memo and updated a running codebook, comparing newly identified codes against those already recorded; these updates were discussed iteratively within the research team (CK–TB). Saturation was achieved when two consecutive interviews (across both participant groups) yielded no substantively new codes and when the emerging thematic structure remained stable during subsequent team review. Considering the relatively focused research question, the homogeneity of the participants, and the richness of the data collected, the sample size was deemed adequate according to the principle of “information power” [31].

The principal investigator (CK) interviewed participants in their homes to create a comfortable, familiar environment conducive to open discussion. All interviews were conducted by the principal investigator (CK), who has prior training in qualitative research methods and experience caring for patients with chronic illnesses. The interviewer had no prior professional or personal relationship with the study participants, thereby minimizing the risk of role conflict or biased responses. Reflexive field notes were kept after each interview to acknowledge and bracket potential preconceptions. In addition, continuous dialogue with a second senior researcher (TB) was used to enhance critical reflection and reduce interviewer bias. The interviews ranged from 40 to 85 min. Field notes were taken immediately after each session to record nonverbal behavior, contextual details, and emerging analytical thoughts. The interview guides for patients and caregivers were developed collaboratively by the research team before ethical approval. The interview guides contain open-ended questions addressing several domains: physical symptoms and impact of the disease, psychological distress, social limitations, spiritual concerns, and unmet needs in the healthcare and support services. The main interview questions for patients and caregivers are provided in the Supplementary Material (Table S1). The central emphasis was on the perceived adequacy of current home-care services, how these services could be improved, and perceived essential interdisciplinary roles that currently do not exist (e.g., physiotherapist, psychologist, counselor).

Patients were invited to describe the evolution of their symptoms and support needs, the day-to-day challenges they encounter, and broader thoughts on quality of life related to having a chronic illness. Caregivers were asked to reflect on the needs of the patients they care for, as these are experienced and managed in everyday caregiving, including patients’ physical, psychological, social, and practical needs. In addition, caregivers were invited to discuss their caregiving responsibilities, emotional strain, and perceived support from formal services, insofar as these factors influenced their ability to recognize and respond to patients’ PC needs. The interview guide was developed as a skeleton to allow flexibility during data collection; however, it was iteratively modified as new themes emerged [32].

In addition to exploring unmet needs, the interview framework also encouraged participants (patients and caregivers) to reflect on how they cope with daily challenges and where they draw emotional or practical strength. This approach was grounded in the holistic philosophy of PC, aiming to capture not only suffering but also the adaptive resources, such as family ties, spirituality, and community support, that enable patients and caregivers to endure and find meaning in the illness experience [21,28,29].

### 2.3. Data Analysis

Thematic analysis was the most suited approach to analyzing the qualitative data in the present study because the method maintains a theoretical flexibility while providing a structured approach to identifying, analyzing, and reporting patterns and themes within qualitative data [32]. Τhematic analysis is particularly geared towards investigations such as our own, which are meant to examine participant experiences and perspectives in specified health and care contexts [33,34]. Our objective was to provide finer detail and deeper insight into the physical, psychological, social, spiritual, and care-related needs of patients with advanced non-malignant chronic conditions and their caregivers.

The interviews were all audio-recorded and transcribed verbatim by the principal investigator, with the original data anonymized. To further protect confidentiality, the presentation order of patients and caregivers in the Supplementary Material is for reporting purposes only and does not indicate fixed dyadic linkage; this approach was adopted to prevent any potential identification of participants or inference of relationships between individuals. The transcriptions were read several times to gain deep familiarity with the data. Two researchers (CK and TB) independently coded the transcripts in NVivo 14 (Lumivero, Denver, CO, USA) following Braun and Clarke’s thematic analysis approach [32]. Coding was primarily inductive, with codes generated directly from the data and iteratively refined through constant comparison across transcripts. Codes were then collated into candidate themes, which were reviewed against the entire dataset and finalized through team discussions to ensure that themes were grounded in and consistently represented by the underlying coded extracts. Themes were then defined and named, with clear descriptions of their scope and boundaries, and illustrated with representative extracts in the final report. The analysis followed an iterative, recursive process, moving back and forth among the phases of data familiarization, coding, and theme development. This non-linear approach ensured that the final themes were deeply grounded in the participants’ narratives, capturing the nuanced experiences of both patients and caregivers. An audit trail was maintained throughout (analytic memos, codebook iterations, and documented decisions), and discrepancies in coding or theme boundaries were resolved through discussion until consensus was reached; if needed, additional senior researchers (CP, EM) were available for adjudication. To enhance methodological rigor and transparency, the study was designed and reported in accordance with established qualitative research quality criteria, including the Consolidated Criteria for Reporting Qualitative Research (COREQ; Table S2) and the Critical Appraisal Skills Programme (CASP; Table S3) checklist. To further enhance transparency and methodological rigor, the primary investigator maintained reflexive journals and analytic memos throughout the study. These records allowed the research team to acknowledge and bracket potential preconceptions, ensuring that the interpretation remained focused on the participants’ lived experiences rather than the researchers’ clinical backgrounds.

During the analysis, iterative steps incorporated new insights and ongoing comparisons across and among interviews to verify internal coherence and thematic integrity [35]. They ensured accuracy through triangulation, since the data were analyzed independently and cross-validated by two members of the research team. Differences in coding or interpretation were discussed until both reached consensus on a single interpretation. The two researchers who undertook the coding had different but complementary scientific backgrounds. The first (CK), a member of the Department of Elderly Care at the Directorate of Social Policy and Public Health of the Municipality of Katerini, has clinical experience in caring for chronically ill patients and specializes in the psychological dimensions of healthcare. The second (TB), a professor with research and teaching experience in qualitative and mixed-methods research and in the quality of life of patients with chronic or life-threatening diseases, contributed to the research process through methodological and theoretical guidance. When the two primary coders could not reach consensus through discussion, two additional senior researchers were available to ensure methodological triangulation and adjudication. Specifically, CP (Assistant Professor in Clinical Psychology with expertise in qualitative methodology) and EM (Professor of Nursing with expertise in the quality of life of older adults) could be consulted to provide an independent perspective on contested codes. Their involvement was planned solely for cases in which consensus could not be achieved between the primary coders. Coding was performed using NVivo 14, which facilitated data organization and theme development. The analysis followed an inductive approach, without a predetermined theoretical framework, with an emphasis on semantic themes, although latent meaning was also identified in some narratives. Although the interview guides included broad prompts about coping and sources of support, resilience/spirituality were not treated as a priori analytic categories; instead, they emerged as recurrent inductive codes and were subsequently interpreted as a cross-cutting dimension linking multiple domains of need. This analytical decision was made during the final stages of theme refinement, as it became evident that resilience was not a standalone category but a dynamic process intersecting with all primary themes. This conceptualization aligns with established qualitative practices for representing complex, interlinked phenomena. For transparency, a sample of the codebook (6–8 main codes with definitions and indicative excerpts) is provided as Supplementary Material (Table S4).

Given the interpretive nature of thematic analysis, considerable attention was paid to preserving each participant’s authentic voice while also seeking common ground across the interviews. Researchers remained mindful of the difficulty introduced by converging individual stories into broader themes, an issue frequently referenced in the literature [32]. To reduce interpretive bias, the findings present examples of outliers and contradictions where appropriate. The final thematic structure was derived through a rigorous, systematic, and transparent analysis. Each step of the data analysis was conducted according to well-established qualitative research practices [33,36] to achieve analytic depth, validity, and interpretive clarity. The analysis was conducted in Greek, the language of the interviews, to preserve cultural and linguistic authenticity. Any part of a quotation presented during the Section 3 was translated into English with close attention to the meaning and context.

Translation process: All interviews were conducted and transcribed in Greek. The quotations included in this paper were translated into English by the principal investigator, who is fluent in both languages and has research experience in qualitative methods. To ensure accuracy, the translated excerpts were reviewed by a second bilingual researcher (TB), and minor adjustments were made to preserve cultural and contextual meaning. Although no formal back-translation procedure was performed, a careful comparison of the original transcripts and the translated excerpts was conducted to ensure fidelity.

## 3. Results

Table 2 depicts the demographic and clinical features of the sample. The study sample includes eight patients and nine caregivers. The median age of patients is 87 years (range: 53 to 92), whereas caregivers are comparatively younger, with a median age of 64 (range: 49 to 84). Most patients were women (87.5%), whereas caregivers were 44.4% men and 55.6% women. When considering marital status, most of the patients are widowed (62.5%), whereas most of the caregivers are married or cohabitating with a common-law partner (66.7%). Among parents, more are in the category of patients with three or more children (62.5%), while caregivers are less clear-cut, with 44.4% also having three or more children. Regarding educational level, 100% of both groups report a level below high school. Most patients are retired (87.5%), while caregivers are mostly retired as well (55.6%); others work (22.2%) or are unemployed (11.1%).

Regarding annual household income, most patients earn between €10,000 and €19,999 (50%), while caregivers are evenly split between less than €10,000 and €19,999 (44.4%) and €20,000–€29,999 (44.4%). The most prevalent chronic conditions for patients include heart failure (62.5%), neurological disease such as amyotrophic lateral sclerosis (ALS), multiple sclerosis, or Parkinson’s disease (50%), and diabetes mellitus (37.5%). Caregivers’ relationships with patients are primarily with other family members (55.6%), followed by spouses/partners (22.2%) and sons/daughters (22.2%). Regarding years of caregiving experience, the majority of caregivers (44.4%) provided care for 4–6 years, while others provided care for 1–3 years (22.2%) or more than 10 years (22.2%). Detailed participant-level characteristics (e.g., duration of illness/caregiving, participant IDs and pseudonyms), as used to identify quotations in the Results, are provided in Table S4 (Supplementary File).

Using reflexive thematic analysis, we identified five overarching themes and sixteen subthemes that capture the multidimensional PC needs of patients with advanced non-malignant chronic conditions and their informal caregivers. Each theme is presented first through an interpretive narrative supported by illustrative quotations. For clarity and readability, a concise table follows each theme, summarizing key points and representative excerpts.

**Figure 1:**
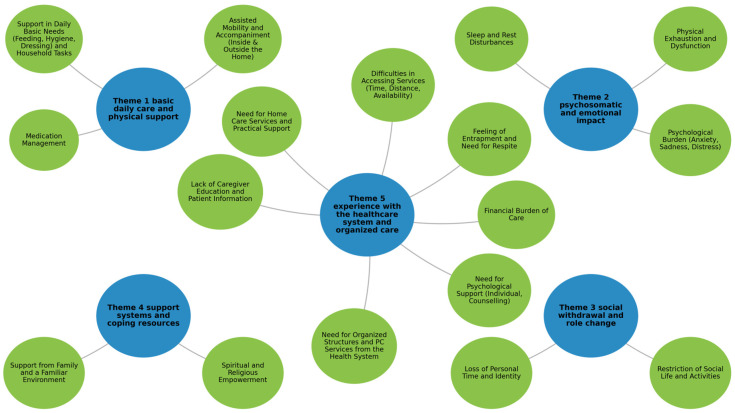
Thematic map illustrating the five themes (shown in blue) and their corresponding subthemes (shown in green).

The five themes were: (1) basic daily care and physical support; (2) psychosomatic and emotional impact; (3) social withdrawal and role change; (4) support systems and coping resources; and (5) experience with the healthcare system and organized care. Figure 1 presents the thematic map showing the five themes and their subthemes. All quotations are attributed using pseudonyms (P-xx for patients and C-xx for caregivers) to ensure anonymity.

### 3.1. Basic Daily Care and Physical Support

Participants underscored a profound and non-negotiable dependence on their caregivers for nearly every aspect of their daily survival. This functional decline was described not merely as a comprehensive loss of autonomy that reconfigures everyday life within the dyad, but also as reshaping the patient’s identity and the caregiver’s role. Across accounts, dependence was expressed as continuous (“all day, every day”) and highly practical, with care needs extending beyond symptom management to the minute-by-minute organization of basic living.

#### 3.1.1. Support in Daily Basic Needs (Feeding, Hygiene, Dressing) and Household Tasks

The data revealed that personal care tasks, such as dressing, bathing, and feeding, have been entirely transferred to informal caregivers, often creating a 24 h cycle of responsibility. As one patient vividly explained:

*“I need help every day. [Name] helps me with dressing, eating, and bathing. I can’t do anything on my own”.*

(P-05)

This transfer was not portrayed as occasional assistance but as full substitution of self-care, with patients framing themselves as unable to perform even minimal daily functions without another person present. For caregivers, this transition is a manual, time-consuming burden that leaves no room for personal respite. While some patients emphasized the need for assistance with personal hygiene, others noted that even basic domestic maintenance had to be entirely managed by others:

*“The household chores, someone else does them”.*

(P-07)

*“It also highlighted the intensity of this role: “As soon as we wake up, […] we have to change her clothes, […] I have to feed her by putting food in her mouth. All of this is our responsibility […]”*

(C-09)

Taken together, these accounts indicate that “basic care” functioned as an all-encompassing routine combining intimate personal care with ongoing household management, sustained primarily by family caregivers.

#### 3.1.2. Medication Management

Safety in pharmacological treatment emerged as a primary concern, with caregivers assuming an informal “clinical monitor” role to prevent omissions and timing or dosage errors. Patients acknowledged that adherence would be difficult to sustain without support, particularly when multiple conditions and treatments had to be coordinated. As one patient explained:

*“I take medication for my blood pressure and diabetes. […] [Name] also helps me, so I don’t forget.”*

(P-04)

Medication routines were described as cognitively demanding and safety-critical, requiring continuous oversight of timing, dosage, and coordination across multiple treatments. Adherence was framed as a task vulnerable to confusion and repetition, particularly as cognitive capacity declined, often requiring the caregiver to take over tracking and error prevention. As one caregiver described:

*“When she needs her medication, I give it to her. I have to remember all the pills, the times, the dosages, because she can’t anymore.”*

(C-02)

For caregivers, this responsibility meant increased cognitive load, as they had to master complex schedules and coordinate the entire regimen on the patient’s behalf. Taken together, these accounts indicate that medication management functioned as an ongoing home-based safety task sustained by caregiver vigilance and cognitive labor.

#### 3.1.3. Assisted Mobility and Accompaniment (Inside & Outside the Home)

The data showed that mobility restrictions were experienced as exhausting and functionally disabling, with participants comparing routine movement inside the home to extreme physical effort and describing everyday ambulation as a constant struggle. Patients emphasized that even minimal distances could exceed their capacity, transforming ordinary transfers into taxing “events” and reinforcing a broader experience of homebound living. As one patient vividly explained:

*“I have difficulty going to the doctor, I can’t move around. […] If I go from here to the other room, it’s like running a thousand meters.”*

(P-03)

Beyond effort, mobility was also portrayed as unsafe due to dizziness and instability, which intensified fear of falls and increased dependence on assistance even for basic walking. One patient described the precariousness of ambulation despite using a walking aid:

*“I can’t walk. With the cane a little, but […] I fall.”*

(P-04)

Consequently, movement outside the home—especially for medical appointments—required meticulous planning and physical preparation by caregivers to manage fragility, prevent symptom escalation, and reduce fatigue. Caregivers described an organized process of pacing and supervision that extended beyond physical help to protective coordination of the whole outing. As one caregiver shared:

*“When we have to go to the doctor, we organize it in advance. I have to prepare her […] and make sure she doesn’t get tired.”*

(C-07)

Taken together, these narratives indicate that mobility support functioned as a combined physical, organizational, and protective task, with caregivers acting as planners and safety monitors to enable movement inside and outside the home.

Additional illustrative quotations for all subthemes are provided in Table 3.

### 3.2. Psychosomatic and Emotional Impact

This theme describes the complex interplay between physical decline and psychological distress, where symptoms are not merely clinical markers but embodied, fluctuating experiences that shape everyday functioning, emotional stability, and the dyad’s capacity to sustain home-based care. Participants often described a “cascade” in which physical fragility intensified fear and vigilance, which in turn amplified distress and disrupted sleep, creating a self-reinforcing cycle for both patients and caregivers.

#### 3.2.1. Physical Exhaustion and Dysfunction

A dominant narrative among patients was profound physical depletion, described as a near-total loss of vitality and a marked reduction in safe, independent movement. Pain, dizziness, and instability were framed as functionally disabling, increasing fear of falls and deepening dependence within the home. Everyday actions were experienced as risk-laden, illustrating how fragility transformed routine mobility into a constant safety concern. One patient described this fragility through an everyday action:

*“I go to drink water holding onto the railings. As soon as I let go, I fall!”*

(P-03)

Caregivers similarly described being affected by the physical consequences of illness, both through continual symptom management and through the cumulative bodily toll of repetitive care. For older caregivers, round-the-clock demands raised concerns about sustainability and their own physical limits. As one caregiver states:

*“What ailments don’t I have? I have diabetes, I have high blood pressure, I have high cholesterol, and muscle pain all over my body. … Yes, yes, alone, from lifting [Name]’s weight.”*

(C-09)

Taken together, these accounts indicate that physical symptom burden was experienced as continuous exhaustion punctuated by episodic crises of instability, requiring constant supervision and contributing directly to caregiver strain.

#### 3.2.2. Psychological Burden (Anxiety, Sadness, Distress)

The data revealed substantial emotional overwhelm alongside the cumulative demands of chronic illness, with participants describing recurrent distress closely tied to uncertainty, loss of control, and the ongoing awareness of decline. Patients portrayed anxiety as intrusive and destabilizing, capturing its intensity in stark language:

*“Anxiety and distress. I feel like I’m going crazy.”*

(P-02)

Sadness was also linked to prolonged immobility and reduced participation in life, presenting as a recurring emotional state rather than a transient reaction. One patient described this as a repeated experience that resurfaced frequently during periods of confinement in bed:

*“I feel a lot of anxiety and sadness being in bed. I feel this way very many times.”*

(P-05)

Caregivers similarly reported persistent worry and emotional fatigue, sometimes accompanied by somatic manifestations of anxiety, reflecting the embodied nature of distress within the caregiving context. As one caregiver shared:

*“I have a rapid heartbeat, pressure on my chest. […] I had a panic attack […] I can’t let myself break down, but my body betrays me.”*

(C-09)

Many participants reported drawing on coping resources—particularly faith/spiritual practices and close family support (further elaborated in Theme 4)—to endure distressing symptoms and emotional strain, suggesting these resources helped them tolerate day-to-day burdens even when formal psychological support was limited. Overall, psychological burden was presented not as an isolated mental-health issue but as an understandable response to sustained physical fragility and caregiving vigilance.

#### 3.2.3. Sleep and Rest Disturbances

The data revealed that sleep was perceived as fragmented and insufficient, characterized by a state of “vigilant rest” shaped by symptoms, anxiety, and fear of adverse events. Patients described repeated awakenings and difficulty returning to sleep, while emotional distress appeared to intensify nighttime arousal and undermine the restorative function of rest. In some accounts, the night was experienced as a period of heightened fear and psychological “darkness,” suggesting that distress was not paused by sleep but continued in a more intensified form. As one patient vividly explained:

*“I saw everything dark! Everything, everything!”*

(P-03)

Patients also linked disturbed sleep directly to the combined burden of anxiety and pain, describing these symptoms as active barriers to rest and recovery. One patient stated:

*“I have anxiety and pain; they make it difficult for me to sleep.”*

(P-08)

Caregivers highlighted an additional psychological barrier to rest: fear of missing a call for help and the perceived responsibility to prevent adverse events, sustaining hypervigilance, and disrupting recovery throughout the night. For some caregivers, repeated night awakenings were embedded in the caregiving routine itself, making sleep a shared, interrupted experience rather than an individual need. For some families, nocturnal vigilance became routinised, turning sleep into a shared caregiving task rather than a restorative period. As one caregiver described:

*“We wake up three times, all night long! […] How can we elderly people endure this?”*

(C-09)

Taken together, these narratives indicate that disrupted sleep functioned as a key pathway through which ongoing symptom burden and caregiving vigilance translated into cumulative dyadic exhaustion.

#### 3.2.4. Feeling of Entrapment and Need for Respite

The data revealed a pervasive sense of confinement, with participants describing themselves as “stuck” within the home environment—an experience that intensified emotional burden and limited opportunities for recovery. This entrapment reflected both physical restriction (mobility limitations and supervision needs) and psychosocial constriction (loss of contact and personal space), reinforcing chronic strain and limiting recovery opportunities. Patients’ accounts suggested that confinement was experienced as a stable condition rather than a temporary phase of illness. As one patient vividly explained:

*“I don’t leave the house; I don’t go anywhere.”*

(P-08)

Caregivers emphasized the lack of even brief respite, noting that uninterrupted supervision eliminated restorative moments and made everyday relief unattainable. The need for constant presence was framed not only as a practical necessity but as a lived experience of continuous tethering, where even a minute of separation felt impossible. As one caregiver stated:

*“[Name] can’t be left alone even for a minute. I can’t even go out to the yard to have a coffee. […]”*

(C-09)

Taken together, these narratives indicate that entrapment amplified distress by compressing both physical space and personal time, thereby sustaining exhaustion and undermining opportunities for recovery.

Additional illustrative quotations for all subthemes are provided in Table 4.

**Table 4:** Psychosomatic and emotional impact.

Subtheme	Quotations
Physical exhaustion and dysfunction	*“I feel constantly tired. I don’t have the strength to do anything. […] not even to lift my hand.” (P-04)* *“I have a lot of pain, especially in my back and legs.” (P-08)* *“Now I had vertigo for 15 days and I couldn’t… I was getting dizzy and about to fall. […]” (C-01)* *“I feel strong psychologically, but my body can’t endure it anymore.” (C-09)*
Psychological burden (anxiety, sadness, distress)	*“Anxiety—so much! … If it gets into your head, you lose it. You go crazy!” (P-03)* *“I get very anxious. I get tired, and I say, ‘why do we have to be like this […]’” (C-01)* *“I feel it when she isn’t well and doesn’t respond… when she doesn’t want to eat, and she doesn’t want to take her medication. That’s what makes me anxious.” (C-04)* *“And then you feel anxious about how you’re going to manage all of this.” (C-04)*
Sleep and rest disturbances	*“My sleep is interrupted. I wake up in the middle of the night, and I can’t sleep well.” (P-04)* *“I don’t sleep well. All night I worry that she might get up and fall.” (C-01)* *“I wake up constantly, every little while. When you’re a caregiver, there is no sleep.” (C-04)* *“If I fall into a deep sleep, I feel guilty if she needs something and I don’t hear her.” (C-07)*
Feeling of entrapment and need for respite	*“I can’t go anywhere. I don’t even have anyone to talk to.” (P-02)* *“I don’t go out at all.” (P-03)* *“I don’t go anywhere either because I can’t. I’m here inside day and night.” (C-01)* *“I don’t want to go out… Why should I have coffee while she’s at home? The coffee doesn’t go down.” (C-09)*

### 3.3. Social Withdrawal and Role Change

The progression of advanced non-malignant chronic conditions was found to trigger a gradual but profound shrinking of the participants’ social universe. This withdrawal was described as an involuntary process where the physical demands of the illness and the burden of caregiving progressively narrowed everyday life to the home, alienating the dyad from their community and previous roles. Importantly, participants framed social withdrawal not only as “less socializing,” but as a sustained loss of contact, reduced participation in meaningful routines, and a sense of being “left behind” by the outside world—often reinforcing loneliness and emotional burden.

#### 3.3.1. Restriction of Social Life and Activities

The data indicated that social restriction unfolded as a cumulative process in which contacts with friends, extended family, and community life progressively weakened. Patients commonly portrayed this as social disappearance—an abrupt reduction in visits and everyday interactions. As one patient vividly explained:

*“I forgot the world! […] they would come maybe once a month.”*

(P-03)

This distancing was not merely a reduction in outings; it also meant minimal interpersonal connection and a persistent sense of isolation at home. Participants repeatedly emphasized that their social sphere had narrowed to the point of having almost no one to engage with beyond the caregiving dyad. As one patient stated:

*“I can’t go anywhere. I don’t even have anyone to talk to.”*

(P-02)

This pattern was mirrored among caregivers, whose own social worlds contracted as caring demands intensified. Caregivers similarly described a constrained social life, often marked by the abandonment of basic social and religious practices that previously provided emotional support and continuity. In several accounts, even church attendance—an important source of connection—was no longer feasible, as it was replaced by remote participation. As one caregiver shared:

*“I don’t visit friends or relatives at all. […] I can’t go to church. We go to church through television.”*

(C-01)

Taken together, these narratives suggest that restricted social participation was experienced as chronic and cumulative, reducing social connectedness and limiting access to informal support systems.

#### 3.3.2. Loss of Personal Time and Identity

The data revealed that, beyond the narrowing of social networks, participants experienced a deeper role transition in which illness and caregiving responsibilities progressively reshaped identity and eroded personal time. Patients associated role change with diminished autonomy and the loss of everyday identities, describing how they could no longer engage in activities that previously sustained meaning, purpose, and self-worth. As one patient vividly explained:

*“I feel sad that I can’t do more than I can’t go to the garden.”*

(P-07)

Caregivers, in turn, portrayed their role as continuous and all-consuming, with limited opportunities for respite, restoration, or independent routine. Their accounts suggested that caregiving became a dominant identity, gradually displacing personal routines and reducing “time for self” to near zero. The need for constant supervision was framed not only as a practical necessity but as a lived experience of continuous tethering, where even a brief pause outside the caregiving space felt impossible. One caregiver described this intensity as follows:

*“[Name] can’t be left alone for even a minute. I can’t even go out to the yard to have a coffee.”*

(C-09)

Overall, these accounts suggest that “role change” involved both a contraction of activities and a redefinition of identity around caregiving and dependence, with limited time for restoration or personal meaning outside the caregiving role. Although social withdrawal intensified loneliness and role strain, dyads often relied on family cohesion and informal support to maintain continuity in daily life, partially compensating for limited formal community services (see Themes 4 and 5).

Additional illustrative quotations for all subthemes are provided in Table 5.

**Table 5:** Social withdrawal and role change.

Subtheme	Quotations
Restriction of social life and activities	*“I can’t go anywhere. […] The children come, but rarely.” (P-04)* *“Old friends come from time to time, but not often.” (P-07)* *“From age 40 to 72, we missed out on all this. […] I can’t even go out for a coffee!” (C-09)*
Loss of personal time and identity	*“I don’t leave the house; I don’t go anywhere.” (P-08)* *“I don’t go anywhere either because I can’t. I’m here day and night, indoors.” (C-01)*

### 3.4. Support Systems and Coping Resources

This theme highlights how dyads sustained home-based care through informal support systems and coping resources, particularly family cohesion, a familiar environment, and spirituality. Participants framed these resources not as “extra positives” but as essential mechanisms enabling them to continue amid the cumulative burden and service gaps described in the preceding themes. In this way, coping resources shaped how needs were experienced and managed across daily care demands, emotional distress, social restriction, and healthcare barriers. At the same time, participants’ accounts suggested that these supports were often finite or unevenly available (e.g., family could help—but not continuously), meaning that coping resources functioned as both a lifeline and a fragile safety net.

#### 3.4.1. Support from Family and a Familiar Environment

The data revealed that the family unit was the cornerstone of palliative care in the Greek community. Participants described close relatives not merely as practical caregivers but as essential emotional anchors who provided security, meaning, and the strength to continue living at home despite progressive decline. Patients articulated this reliance in absolute terms, framing family support as the condition that makes home-based care possible. As one patient vividly explained:

*“[Name] helps me. If I didn’t have him, I would be finished.”*

(P-02)

At the same time, support was portrayed as meaningful but not limitless—often bounded by availability and competing obligations. Some patients described family help as substantial but necessarily intermittent, reflecting work demands and other household responsibilities. One patient illustrated this balance:

*“The children help me, but they can’t be here all the time. [Name] takes care of me as much as he can.”*

(P-04)

This reliance was mirrored among caregivers, who framed the presence of close relatives as a crucial source of security—sometimes less as “help with tasks” and more as the reassurance of not being alone in responsibility. As one caregiver stated:

*“My daughter is my support. […] I know she’s here.”*

(C-09)

Taken together, these accounts indicate that family support operated as both a practical and emotional infrastructure that sustained home-based care, while the familiar home environment further reinforced stability and continuity by preserving routines, relationships, and meaning despite decline.

#### 3.4.2. Spiritual and Religious Empowerment

The data revealed that spirituality functioned as a pervasive coping strategy, providing meaning, comfort, and emotional grounding in the face of suffering and uncertainty. For many participants, prayer was described as a constant, internal dialogue that helped them endure when physical medicine and formal psychosocial support felt insufficient. As one patient vividly explained:

*“I pray constantly! […] It helps me!”*

(P-03)

At the same time, spiritual coping was not uniformly experienced as comforting. Some participants expressed uncertainty or a lack of relief despite prayer, indicating variability in how religious practice translated into emotional benefit and suggesting that spiritual engagement did not always resolve distress. As another patient stated:

*“I pray, but I don’t know if it helps me. I don’t feel relief.”*

(P-04)

For caregivers, faith was also articulated as a foundational meaning system that fostered acceptance and endurance, functioning as a shared mechanism of resilience within the dyad. As one caregiver noted:

*“I know that whatever the Lord and the Virgin Mary say will happen.”*

(C-09)

Taken together, these accounts indicate that spiritual and religious practices operated as meaning-based coping resources that could buffer distress, often substituting for structured psychological support that was frequently inaccessible, while also reflecting unmet psychosocial needs when emotional relief remained limited.

Additional illustrative quotations for all subthemes are provided in Table 6.

**Table 6:** Support systems and coping resources.

Subtheme	Quotations
Support from family and a familiar environment	*“… helps me constantly. If it weren’t for her, I don’t know what I would do.” (P-05)* *“My daughters-in-law and my children help me as much as they can.” (P-08)* *“If we want to go to a doctor, the children will take us.” (C-01)*
Spiritual and religious empowerment	*“I go to church, I confess, and this helps me a lot.” (P-06)* *“I would like it if a priest came to the house for holy oil blessing.” (P-07)* *“We believe deeply. We pray and say, ‘My God, let us be well’.” (C-01)*

### 3.5. Experience with the Healthcare System and Organized Care

Participants described their interaction with the healthcare system as a fragmented and stressful experience, characterized by significant barriers to access and a profound lack of specialized support for non-malignant conditions. Across accounts, participants described a reactive rather than proactive system, with limited continuity, scarce home-based provision, and substantial reliance on families to “hold care together” between appointments and crises.

#### 3.5.1. Need for Home Care Services and Practical Support

The data revealed a strong, near-unanimous call for home-based clinical and practical services to alleviate the daily physical burden of advanced illness and sustained caregiving. Patients emphasized that even minimal professional input—particularly physiotherapy and nursing—could improve mobility, functioning, and overall safety at home. As one patient vividly explained:

*“A physiotherapist would help me to move a little.”*

(P-02)

This need extended beyond physiotherapy to broader practical/home support, with delayed approvals meaning that formal assistance often arrived **after** functional decline had already progressed. One patient highlighted the ongoing wait for formal approval, noting that the needed supports were not readily available even as functional decline was already advanced.

*“I’m waiting for my personal assistant to be approved […]”*

(P-06)

Caregivers echoed these requests and framed the absence of professional home care as a significant safety concern, describing the need for continuous coverage and additional personnel to ensure safe supervision—needs not met within standard community services. As one caregiver stated:

*“A girl… and two 24-hour [caregivers] to be there to take care of [Name]. […]”*

(C-09)

Taken together, these accounts indicate that home care services and practical support were perceived as essential “missing pieces” for sustaining home-based care, reducing caregiver strain, and preventing avoidable deterioration or crises.

#### 3.5.2. Difficulties in Accessing Services (Time, Distance, Availability)

The data revealed that navigating the public health sector was often experienced as a bureaucratic struggle, in which delays, limited availability, and distance-related barriers compounded symptom burden and caregiving strain. Participants described difficulty securing even basic medical services, such as prescriptions, indicating that continuity of treatment could be disrupted by shortages in access. As one patient vividly explained:

*“We can’t find a doctor to prescribe the medications. […]”*

(P-03)

For some patients, restricted mobility made access itself a significant obstacle, amplified by long waiting times and the physical inability to travel. Patients described prolonged delays in hospital scheduling as an additional layer of burden on top of their functional limitations. As one patient stated:

*“It’s difficult to go to the doctor; I can’t move around. […] Appointments at hospitals take too long.”*

(P-08)

Caregivers echoed these experiences and emphasized the system’s slow response as a direct threat to timely care, noting that appointments were postponed for months and leaving families to manage symptoms and risks in the interim. As one caregiver noted:

*“We make an appointment at the hospital, and they tell us ‘Come back in three months. […]’”*

(C-09)

In addition to delays, caregivers described service complexity and gaps in provision, shaped by evolving comorbidities and increasing care needs, which intensify the difficulty of navigating fragmented services. A recurring perception was that urgent support was simply unavailable, leaving families feeling abandoned by the system and forced to rely on themselves during crises.

#### 3.5.3. Financial Burden of Care

The data revealed that the financial strain of managing advanced chronic illness at home was a pervasive source of insecurity, shaping what families could realistically sustain in daily care. Participants described pensions as insufficient to cover escalating costs (e.g., medications, medical supplies, transportation, and, when needed, private assistance). Patients emphasized the lack of formal financial support as part of the burden, framing this absence of state assistance as an additional vulnerability of home-based care. As one patient noted:

*“We don’t have financial help from the state.”*

(P-03)

Caregivers described how constrained household budgets made it difficult to pay for additional help, even when the need for respite or practical support was clear. The inability to hire assistance was framed as a forced trade-off rather than a preference, reflecting the limits of family resources. As one caregiver explained:

*“Look, even help […] I could hire a woman […]”*

(C-09)

Taken together, these narratives indicate that financial burden operated as a structural constraint on care, directly shaping access to medications, services, and additional support, and amplifying stress and uncertainty for both patients and caregivers.

#### 3.5.4. Lack of Caregiver Education and Patient Information

The data revealed a significant gap in guidance and structured information, with caregivers describing how they were frequently left to manage complex clinical tasks through trial and error. This absence of training intensified emotional strain and uncertainty about patient safety, as families assumed responsibility for symptom monitoring and day-to-day clinical decisions without preparation or supervision. Patients also recognized this informational deficit, describing home-based care as an improvised learning process. As one patient explained:

*“There’s no one to show us what to do. We learn everything on our own.”*

(P-04)

Caregivers echoed this experience, portraying caregiving competence as something acquired “in the moment,” through repeated practice rather than a supported transition. As one caregiver stated:

*“There’s no training for caregivers; we learn everything on our own, through practice.”*

(C-08)

Taken together, these accounts indicate that inadequate caregiver education functioned as a structural gap in community care, amplifying distress and heightening perceived risk in home-based management.

#### 3.5.5. Need for Organized Structures and PC Services from the Health System

The data revealed a clear and consistent call for integrated PC models and specialized services that are currently absent in the local context. Participants described the existing system as fragmented and reactive, leaving families to coordinate care on their own and manage needs without a stable support framework. Many compared this reality with more organized international models and expressed a desire for institutional structures similar to those available elsewhere in Europe. As one caregiver explained:

*“To establish institutions as they have in Europe. […]”*

(C-09)

Participants also emphasized the need for more regular clinical monitoring and proactive follow-up, rather than episodic or crisis-driven contact. A patient expressed the need for increased professional presence and ongoing assessment:

*“I would like doctors to come more often, to monitor us. […]”*

(P-04)

Taken together, these accounts indicate that calls for organized PC structures reflect both practical gaps in service delivery and a broader desire for continuity, coordination, and shared responsibility across the health system.

#### 3.5.6. Need for Psychological Support (Individual, Counseling)

The data revealed that professional psychological support was perceived as a critical but neglected component of care. Participants described a desire for a space to process emotional distress, particularly in the context of prolonged uncertainty, functional decline, and sustained caregiving vigilance. Patients often framed this need in relational terms—as the wish to talk with someone who could listen and help them manage their emotional burden. As one patient vividly explained:

*“It would help me to have someone to talk to.”*

(P-02)

Caregivers likewise acknowledged the potential value of counseling. Still, they emphasized that the intensity of caregiving responsibilities left little or no time for self-care, turning psychological support into an “unreachable” resource even when perceived as beneficial. As one caregiver stated:

*“We would like to talk to a psychologist, but we don’t have time even for that.”*

(C-01)

Despite financial strain and fragmented access to services, participants often reported persisting with home-based care by mobilizing informal support and meaning-based coping resources, which sometimes masked unmet needs while simultaneously sustaining care continuity (see Theme 4). In this context, the perceived absence of coordinated services and timely professional input appeared to intensify reliance on family-led care and contribute to delayed escalation until needs became unmanageable. Taken together, these narratives indicate that the need for psychological support was widely recognized but was structurally constrained by limited access and, critically, by a lack of time within the caregiving routine.

Additional illustrative quotations for all subthemes are provided in Table 7.

**Table 7:** Experience with the healthcare system and organized care.

Subtheme	Quotations
Need for home care services and practical support	*“If a physiotherapist could come to the house, it would help a lot.” (P-03)* *“It would help me if someone came to the house to help me. […]” (P-04)* *“It would help to have physiotherapy at home. […]” (C-01)* *“I would like someone to help for a few hours—not 24-hour care, but a few hours, even just for company and support.” (C-02)*
Difficulties in accessing services (time, distance, availability)	*“Maybe the ‘Help at Home’ service could come more often… and [someone] for physiotherapy. I mean for free—so you don’t have to pay.” (P-07)* *“If someone could come to the house—something like ‘Help at Home’.” (P-08)* *“We have difficulties because I’m now diabetic […]” (C-01)* *“There’s no provision/care for us. If we need something urgently, we’re on our own.” (C-08)*
Financial burden of care	*“My pension isn’t enough for medications and doctors.” (P-08)* *“The money doesn’t stretch—my medications, the little supplies, the catheters; we buy all of these with our own money.” (C-03)* *“The pension isn’t enough even for medications. […]” (C-08)*
Lack of caregiver education and patient information	*“I would like more medical guidance—doctors coming to the house.” (C-04)* *“Never, never. We just did whatever we felt was right—by instinct.” (C-09)* *“They leave us alone, without any training, without anyone to show us what to do.” (C-09)*
Need for organized structures and PC services from the health system	*“If there was an organized structure that we could turn to, […]” (C-08)* *“We need care centers for these people, because we can’t do everything on our own.” (C-05)*
Need for psychological support (individual, counseling)	*“I don’t know if a psychologist would help me; I haven’t tried.” […] (P-08)* *“I know that if I go to the psychologist again, they will support me, but I don’t have time to go.” (C-09)*

## 4. Discussion

This qualitative study delineates a multidimensional framework of needs and experiences among patients with advanced non-malignant chronic conditions and their caregivers, as derived from thematic analysis. Five central themes were identified: (1) basic daily care and physical support, (2) psychosomatic and emotional impact, (3) social withdrawal and role change, (4) support systems and coping resources, and (5) experience with the healthcare system and organized care. Overall, our findings both align with and refine existing evidence on patient and caregiver needs in PC, while highlighting context-specific implications for community-based care models [12,13,14,37,38]. Importantly, we interpret coping and resilience-related processes as embedded across domains of need (rather than as a separate “positive” outcome), which helps clarify how dyads sustain home care amid high burden and service gaps.

### 4.1. Theme 1: Basic Daily Care and Physical Support

Participants described extensive dependence in core activities of daily living and a pronounced transfer of care tasks to family caregivers (feeding, hygiene, dressing, medication routines, and mobility support). International literature similarly documents high symptom burden and functional dependence among people with advanced non-malignant chronic conditions—often comparable to, or exceeding, cancer populations [12,17,21]. Our findings extend this evidence by specifying how dependence is operationalized in everyday home routines and by illustrating the practical “task load” borne by caregivers in the absence of systematic professional back-up. In the Greek community context, these accounts suggest that support needs extend beyond clinical symptom control to include practical assistance (e.g., safe mobilization and basic care) that helps maintain stability at home. Accordingly, service planning should prioritize integrated home-based support—including home physiotherapy where indicated, basic nursing monitoring, and medication management support—to mitigate preventable deterioration and reduce avoidable service use, consistent with evidence on home-oriented interventions and quality-of-life outcomes [28,29,39]. In addition, the intensity and continuity of care described here reinforce the need to assess caregiver capacity as a safety-critical component of home-based PC planning, not merely as an “adjunct” consideration.

### 4.2. Theme 2: Psychosomatic and Emotional Impact

A second key finding concerns the coupling of physical distress (pain, fatigue, vertigo, functional decline) with psychological strain (anxiety, sadness, panic-like episodes) and persistent sleep disruption. Prior evidence has emphasized the psychological burden associated with chronic illness trajectories and caregiving roles [23]. Our analysis adds clarity by indicating a plausible mechanism through which burden escalates symptom instability and functional fragility drives continuous vigilance, which in turn fragments sleep and undermines caregiver recovery, amplifying distress and reducing coping capacity. In the results, physical fragility was frequently framed in terms of fall risk and loss of bodily control (e.g., needing to “hold onto railings” to move safely), alongside persistent pain (e.g., back/leg pain) and prolonged episodes of vertigo reported within the dyad. This pattern supports the need to view psychosomatic distress and sleep disturbance as core targets within community PC, not only as “secondary” consequences. The practical implication is that symptom management strategies should be paired with accessible psychosocial support and structured respite options, given that respite interventions are reported to be effective and may buffer caregiver depletion [40]. Consistent with the Results, dyads frequently drew on coping resources (e.g., family support and spirituality) to “hold distress together,” suggesting that psychosomatic burden cannot be fully understood without considering the everyday coping ecology within which symptoms are managed. Notably, psychological burden in the results was not limited to generalized “anxiety”; participants described acute affective escalation and existential distress (e.g., feeling that “everything turned dark”), repeated sadness associated with prolonged bedbound states, and caregiver emotional exhaustion (“why do we have to be like this”), which together suggest fluctuating intensity rather than a static level of distress.

Sleep disruption was similarly multi-determined: beyond physical symptoms (pain/anxiety interfering with sleep), caregivers reported anticipatory worry about falls and night-time safety, which reinforced a pattern of “vigilant rest” that maintained arousal and undermined recovery. Taken together, these accounts support targeting (i) symptom control linked to falls/instability, (ii) sleep protection strategies, and (iii) psychological support that is realistically accessible within home-based care pathways.

### 4.3. Theme 3: Social Withdrawal and Role Change

Participants described a progressive decline in social participation and meaningful activities, accompanied by role changes and the erosion of personal time and identity, particularly among caregivers. Similar social restrictions and role strain have been described as key contributors to reduced quality of life in chronic illness contexts [28,30,41]. Our findings underscore that social withdrawal is not merely an “outcome” of illness but also a sustaining condition that can intensify psychological vulnerability and reduce the availability of informal support. In the results, withdrawal was expressed in concrete terms of social disappearance and infrequent contact (e.g., being visited “maybe once a month”, family coming “rarely”, and friends coming “from time to time, but not often”), suggesting a transition from participation to sporadic, low-intensity interaction.

In Greek community settings, where formal day-center options and structured social reintegration pathways may be limited, these results indicate a need to link community and municipal services to social and practical supports (e.g., facilitated support groups, day-care options when available, and social prescribing approaches). Such initiatives can protect identity and social connectedness and may indirectly support the sustainability of caregiving. Caregiver accounts also conveyed a “life-course” sense of loss (e.g., “from age 40 to 72, we missed out on all this”), indicating that role captivity can accumulate over years and reshape identity beyond the immediate demands of care.

Moreover, the data suggest that when social worlds shrink, dyads may rely more heavily on “closed” family-based coping (Theme 4), which can sustain care continuity but may also increase the risk that unmet needs remain hidden. This is reinforced by descriptions of continuous home confinement (“day and night, indoors”) and curtailed spiritual/community participation (e.g., attending church via television), highlighting the dual loss of social connection and meaning-making spaces.

### 4.4. Theme 4: Support Systems and Coping Resources

Participants frequently emphasized family ties, a familiar environment, and spiritual/religious practices as coping resources and sources of emotional stability. Prior literature similarly highlights the protective role of social connection and spiritual resources in coping with chronic illness and caregiving strain [11,13]. However, our findings refine this relationship by showing that informal support often functions as both a buffer and a necessity: it provides essential care capacity when professional services are limited, yet it may also conceal unmet needs by normalizing high caregiver burden. In practice, community PC approaches should recognize family networks as key partners while ensuring that reliance on informal support does not substitute for formal care. This includes communication practices that proactively assess caregiver capacity and needs, and service designs that facilitate timely linkage to supports. Crucially, these coping resources may have a dual function: while sustaining endurance and continuity of home care, they may also raise thresholds for help-seeking and delay escalation to formal services.

Taken together, our findings suggest that these coping resources operate across domains of need, helping dyads sustain home care amid intensive daily care demands (Theme 1), psychosomatic distress and sleep disruption (Theme 2), social withdrawal and role change (Theme 3), and responses to healthcare barriers and fragmented services (Theme 5). Accordingly, coping can be understood as a relational, meaning-based process embedded in everyday home care, which may help explain both the persistence of home-based care despite a high burden and the tendency to delay escalation to formal support until accumulated unmet needs become crisis-driven.

### 4.5. Theme 5: Experience with the Healthcare System and Organized Care

Participants described structural barriers, including difficulty accessing services (waiting times, distance, availability, prescription medications), financial strain, and a lack of caregiver training and patient information. While service fragmentation is a recurrent challenge across systems, our findings highlight how these barriers compound symptom distress and caregiving strain, reinforcing vulnerability across non-cancer trajectories [12,13,14]. In the Greek context, these accounts are particularly salient given the limited availability of specialized PC services and the reliance on community/municipal support structures [2,27]. Consistent with the results, participants portrayed care as largely episodic and reactive rather than proactive, with limited continuity and few home-based follow-up options—creating “gaps” in which families must manage on their own until needs escalate.

The implication is twofold: first, improving access requires clearer community-based pathways and coordination mechanisms; second, caregiver education should be treated as a core service component, consistent with evidence that caregiver training improves care processes and caregiver wellbeing [25,42]. Importantly, caregiver preparedness is also a determinant of patient safety and service use: limited training may compromise symptom monitoring and medication adherence, increasing the likelihood of avoidable crises and unplanned healthcare utilization. These findings support framing family caregivers as co-providers within the home care plan, requiring structured training, psychosocial support, and respite as integrated components rather than optional add-ons.

In addition, the results highlight specific system “pressure points” that shape lived experience: delays in accessing or approving formal home assistance (e.g., personal assistants), long waiting times for appointments despite mobility constraints, and limited availability of timely prescriptions or urgent advice. Participants also emphasized a need for more regular clinical monitoring and home-based follow-up, suggesting that proactive review (rather than crisis-driven contact) could reduce instability and caregiver vigilance. Finally, the persistence of home-based care despite system gaps suggests that coping resources (Theme 4) can function as a “bridge” that sustains continuity—yet may also mask cumulative unmet needs until a crisis forces contact with acute services.

### 4.6. Practical and Policy Implications for Community and Home-Based PC

Taken together, the findings indicate priorities for clinical practice, service planning, and policy, particularly for non-cancer trajectories in community settings. First, themes related to functional dependence and service barriers (Themes 1 and 5) support strengthening integrated home-based support, including coordinated symptom review, mobility/physiotherapy support where indicated, and medication management assistance [28,29,39]. Second, psychosomatic distress, sleep disruption, and social restriction (Themes 2 and 3) underscore the importance of embedding psychosocial support and respite options within community pathways, rather than treating them as ancillary services [40]. Third, the caregiver-focused findings (Themes 1, 2, and 5) indicate that caregiver assessment, structured education, and ongoing support should be considered core components of home-based PC models, consistent with international evidence on the value of caregiver training [25,42]. In Greece, where specialized PC provision remains limited, these priorities reinforce the need for coordinated models spanning primary care, hospital outreach, and municipal community services, such as “home help” programs, to reduce fragmentation and enhance continuity of care [2,27]. In addition, a “coping-aware” service design is needed: clinicians should explicitly assess whether reliance on family/spiritual coping is sustaining care in the absence of adequate services and proactively offer escalation pathways and respite before distress becomes crisis-driven. Given the results, this also implies shifting from episodic/case-by-case responses to proactive follow-up and continuity mechanisms (e.g., scheduled home review/monitoring, rapid advice pathways), so that “urgent gaps” do not default to family-only management.

### 4.7. Strengths, Limitations, and Methodological Considerations

This study benefited from data collection in participants’ familiar environments, which supported the depth and authenticity of accounts. The thematic analysis was conducted with methodological rigor aligned with theoretical approaches [32], and informed by established guidance on analyzing and presenting qualitative data [34,36]. Conducting analysis in Greek preserved cultural and linguistic nuance and reduced the risk of meaning loss through translation. Nevertheless, transferability may be limited by contextual specificity: the sample was geographically concentrated and shaped by a single municipal service context, resulting in a predominantly older and lower-education profile. These features reflect the population served rather than an absence of sampling variation, but they should be considered when interpreting transferability to other settings, consistent with qualitative research standards [36,43]. Selective participation bias is possible, and end-of-life stage experiences were not systematically represented, reflecting recruitment pathways rather than explicit exclusion. Finally, because coping resources are culturally embedded, the prominence of family cohesion and spirituality should be interpreted within the Greek sociocultural context; future work should examine how these patterns vary across regions and service configurations.

### 4.8. Suggestions for Future Research

Further qualitative research with larger, more diverse samples is needed to explore transferability across regions and service contexts. In addition, larger multicenter quantitative studies could evaluate the impact of targeted interventions (e.g., caregiver education, home-based physiotherapy/nursing supports, and respite structures) on quality of life, caregiver outcomes, and healthcare utilization [10]. Finally, the development and validation of needs assessment tools tailored to advanced non-malignant chronic conditions may support standardized referral criteria and monitoring of service effectiveness, in line with the study’s overarching aims [12]. Given the observed “coping-and-burden” interplay, future studies should also examine whether strengthening formal services reduces the extent to which coping resources “mask” unmet needs and delay timely palliative support.

## 5. Conclusions

This study provides an in-depth community-based qualitative account of the PC-related needs and experiences of Greek patients with advanced non-malignant chronic conditions and their caregivers. The findings highlight interconnected domains of functional dependence and intensive daily care demands; psychosomatic distress and sleep disruption; social restriction and role change; reliance on informal support and spirituality; and persistent gaps in access, training, and organized care. Crucially, the study identifies coping resources and resilience-related processes—grounded in spirituality and family solidarity—as a cross-cutting, relational mechanism that mediates how these needs are navigated in daily life and can, at times, sustain home care while simultaneously obscuring cumulative unmet needs when formal support is limited.

Three key implications emerge: First, for non-cancer trajectories, community- and home-based PC should prioritize integrated home support that addresses both clinical and practical needs, including mobility and medication management, where indicated. Such interventions must account for the 24 h cycle of responsibility often borne solely by informal caregivers. Second, family caregivers should be recognized as co-providers within home care pathways, supported through structured education, psychosocial support, and respite options to sustain caregiving capacity and reduce avoidable crises. Recognizing caregivers’ preparedness is essential, as their own well-being directly impacts the sustainability of home-based care. Third, policy and service planning should strengthen coordination across primary care, hospital outreach, and municipal community services to reduce fragmentation and improve continuity of care in settings with limited specialist PC provision. Service models should proactively leverage existing resilience resources through strengths-based, “coping-aware” assessment and communication practices, while ensuring that formal professional support remains accessible to prevent caregiver depletion and avoid crisis-driven escalation to acute services. Future research should test targeted community-based interventions and advance the development of condition-appropriate needs assessment tools to improve equitable access and quality of care for people living with advanced non-malignant chronic conditions and their families, and to evaluate whether strengthening formal provision reduces the extent to which informal coping resources “mask” unmet need over time.

## Figures and Tables

**Table 1:** Inclusion and exclusion criteria for patients and caregivers.

Group	Inclusion Criteria	Exclusion Criteria
Patients	Diagnosed with advanced non-malignant chronic diseases (e.g., COPD, CHF, renal failure, neurological conditions such as ALS or MS), provided they retain sufficient communication abilities to engage in an interview.	Diagnosed with any cancer
	Enrolled in the “Help at Home *” program	
	Age 18 years and older	Severe psychopathology
	Adequate communication skills and sufficient knowledge of Greek to participate in interviews or questionnaires	Diagnosed with neurological or psychiatric conditions that severely impair communication and thus prevent meaningful participation in interviews
	Provided informed consent	
	No prior professional relationship between the researcher and the patient or their family	
Caregivers	Primary caregiver during the patient’s illness (e.g., family member or close friend).	Professional caregivers or paid caregivers
	Age 18 years or older	Individuals with current or prior employment in healthcare professions
	Adequate communication skills and sufficient knowledge of Greek to participate in interviews	Severe psychopathology

Note *: The “Help at Home” program is a government-funded community service that provides in-home care to older adults and people with chronic illnesses. Ordinary members of the interdisciplinary team include social workers, nurses, and home care aids.

**Table 2:** Demographic and clinical characteristics of the participants.

Participant Characteristics	Patients (*N* = 8)	Caregivers (*N* = 9)
	*n* (%)	*n* (%)
Age in years, median (range)	87 (53–92)	64 (49–84)
Gender		
Male	1 (12.5)	4 (44.4)
Female	7 (87.5)	5 (55.6)
Marital status		
Married or common-law partner	2 (25)	6 (66.7)
Separated or divorced	0	2 (22.2)
Single	1 (12.5)	1 (11.1)
Widowed	5 (62.5)	0
Children		
No	1 (12.5)	1 (11.1)
Yes, one child	0	2 (22.2)
Yes, two children	2 (25.0)	2 (22.2)
Yes, three or more	5 (62.5)	4 (44.4)
Education		
College, University, or higher education	0	0
High school	0	0
Below high school (including incomplete elementary)	8 (100)	9 (100)
Employment status		
Retired	7 (87.5)	5 (55.6)
Employed	0	2 (22.2)
Unemployed	0	1 (11.1)
On disability	1 (12.5)	1 (11.1)
Annual household * income		
Less than €10,000	2 (25)	1 (11.1)
€10,000–€19,999	4 (50)	4 (44.4)
€20,000–€29,999	1 (12.5)	4 (44.4)
€30,000–€39,999	1 (12.5)	0
Chronic Disease/Condition (patient)		
Chronic Respiratory Diseases (e.g., COPD, Chronic Respiratory Failure, Idiopathic Pulmonary Fibrosis)	1 (12.5)	
Heart Failure	5 (62.5)	
Diabetes Mellitus	3 (37.5)	
Chronic Organ Diseases (e.g., Chronic Kidney Disease, Liver Cirrhosis)	1 (12.5)	
Neurological Diseases (ALS, Multiple Sclerosis, Parkinson’s Disease)	4 (50)	
Dementia (Alzheimer’s Disease or other forms)	1 (12.5)	
Autoimmune Diseases (e.g., Rheumatoid Arthritis, Systemic Lupus Erythematosus)	1 (12.5)	
Chronic Endocrine Disorders (e.g., Hypothyroidism)	2 (25)	
Chronic Pain (e.g., Fibromyalgia)	1 (12.5)	
Relationship with patient (caregiver)		
Spouse or partner		2 (22.2)
Son or daughter		2 (22.2)
Other family members (e.g., siblings, grandparents, aunts/uncles)		5 (55.6)
Years of patient care (caregiver)		
<1 year		0
1–3 years		2 (22.2)
4–6 years		4 (44.4)
7–10 years		1 (11.1)
>10 years		2 (22.2)

* Note: Household income refers to the total annual income earned by all individuals living together in the same household and sharing expenses.

**Table 3:** Basic daily care and physical support.

Subtheme	Quotations
Support in Daily Basic Needs (Feeding, Hygiene, Dressing) and Household Tasks	*“[Name] does everything for me.” (P-02)* *“I can’t write; I can’t eat by myself. Mom and dad help me with everything.” (P-06)* *“I’ll bring him water to take his pills, […] I’ll dress him, I’ll take him to the toilet […]” (C-01)*
Medication Management	*“I get confused with all the different tablets I have for my lungs and my heart. If my daughter didn’t prepare the weekly dispenser for me, I would definitely take the wrong dose.” (P-03)* *“Medication is vital. If she doesn’t take it on time, she might not feel well. I have to monitor everything.” (C-05)*
Assisted Mobility and Accompaniment (Inside & Outside the Home)	*“I can’t walk; I get dizzy…” (P-02)* *“I’ve been in a wheelchair for 20 years. […] I need help.” (P-06)* *“Transportation is difficult, especially when there’s no one to help us. We try to manage on our own.” (C-04)*

IN ALL Table 3, Table 4, Table 5, Table 6 and Table 7: “… signifies an incomplete sentence by the participant, while […] denotes sections of the interview omitted by the authors for brevity; [name] = anonymized name”.

## Data Availability

The data generated and analyzed in this study (de-identified interview transcripts) are not publicly available due to privacy and ethical restrictions, but are available from the corresponding author upon reasonable request and with appropriate ethical approval.

## Supplementary Materials

The following supporting information can be downloaded at https://www.mdpi.com/article/10.3390/healthcare14040479/s1. Table S1. Main semi-structured interview questions for patients and informal caregivers; Table S2: COREQ (Consolidated Criteria for Reporting Qualitative Research) checklist; Table S3: CASP Qualitative Checklist; Table S4. Excerpt of codebook and theme development; Table S5: Individual demographic and clinical profiles of patients and their primary informal caregivers (pseudonyms).

## Abbreviations

The following abbreviations are used in this manuscript:

PC	Palliative Care
COPD	Chronic Obstructive Pulmonary Disease
ESRD	End-Stage Renal Disease
CHF	Congestive Heart Failure
ALS	Amyotrophic Lateral Sclerosis
MS	Multiple Sclerosis
