# Supplementary material for: Palliative Care Needs in Advanced Non-Malignant Chronic Conditions: A Qualitative Study of Greek Patients’ and Caregivers’ Perspectives
Source: Healthcare (Basel). 2026 Feb 13;14(4):479. doi: 10.3390/healthcare14040479 (PMC12940276; doi:10.3390/healthcare14040479)
Supplement: Supplementary file 1 [file healthcare-14-00479-s001.zip › healthcare-4087758-supplementary.pdf]

Supplementary material

Table S1. Main semi-structured interview questions for patients and informal caregivers.

**Domain**	**Patients (examples of main questions)**	**Informal caregivers (examples of main questions)**
Background / context	1) Can you tell me a little about yourself (e.g., age, living situation)?<br>2) What is your main chronic condition(s), and how long have you been living with it/them?<br>3) How would you describe the overall course of your illness so far?	1) Can you tell me a little about yourself (e.g., age, living situation)?<br>2) What is your relationship to the patient, and how long have you been providing care?<br>3) What does your caregiving role typically involve day-to-day?
Physical symptoms & daily burden	4) What are the main symptoms you deal with on a day-to-day basis (e.g., breathlessness, pain, fatigue)?<br>5) Which symptoms are the most difficult to manage and why?6) What makes symptoms worse or better at home?	4) What are the patient's main needs and symptoms that you help with most often?<br>5) Which symptoms or situations are the most difficult for you to manage at home, and why?<br>6) What makes symptoms worse or better at home?
Functioning / ADLs & mobility	7) How often do you need help with everyday activities (e.g., dressing, bathing, moving around, medications)? What kind of help?<br>8) How would you describe your current energy level and mobility/functioning?	7) How often does the patient need help with everyday activities (e.g., dressing, bathing, moving around, medications)? What kind of help?<br>8) How would you describe the patient's current energy level and mobility/functioning?
Psychological needs	9) Do you experience anxiety, sadness, or low mood related to your illness? Can you describe how this affects you?<br>10) What worries you most at this stage of your illness?	9) Do you experience anxiety, exhaustion, or emotional strain because of caregiving? Can you describe it?<br>10) What worries you most at this stage of the patient's illness?
Social needs & participation	11) Have you had to limit your social activities or relationships? If yes, what are the main reasons?<br>12) What kinds of social support/services or activities would help you (e.g., support groups, home visits, practical assistance)?	11) Have you had to limit your own personal or social activities because of caregiving? If yes, how?<br>12) What kinds of social or practical support would make caregiving more sustainable for you?
Services & unmet needs in care	13) What support/services do you currently receive at home (including "Help at Home")? How adequate is it for your needs?<br>14) What additional support or professional roles would you consider important but	13) Which formal services have been most helpful in caring for the patient?<br>14) Where do you see gaps in current services/support, and what would you like to be available?

	currently missing (e.g., physiotherapist, psychologist, counselor)?	
Coping resources / resilience (holistic PC)	15) When things are difficult, where do you draw strength or practical/emotional support from (e.g., family, faith, community)?<br>16) Are there any personal strategies that help you manage daily challenges at home?	15) Where do you draw emotional or practical strength from in caregiving (e.g., family, faith, community)?<br>16) Do you feel you have enough support for your own well-being? What would help most?
Spiritual concerns (where applicable)	(If raised by participant)<br>17) Are there any spiritual/religious concerns that matter to you in relation to your illness and care? What support would you want?	(If raised by participant)<br>17) Are there any spiritual or religious concerns related to caring for the patient (e.g., meaning, fear, need for rituals)?<br>18) Would you like support in this area, and what kind?

Note: Questions were used flexibly; additional prompts were applied as needed to elicit depth and clarify participants' experiences.

Table S2. COREQ (Consolidated Criteria for Reporting Qualitative Research) Checklist.

Domain / Item	Guide Questions / Description	Reported in Manuscript
**Domain 1: Research team and reflexivity**		
1. Interviewer/facilitator	Which author conducted the interview?	PI (CK) conducted all interviews (Methods: Qualitative interviews).
2. Credentials	What were the researcher's credentials?	CK: RN, MSc, PhD(c), Dipl. Psych.; TB: Professor, PhD, MSc, RN, Dipl. Psych.
3. Occupation	What was their occupation at the time of study?	CK: Department of Elderly Care, Municipality of Katerini; TB: Professor, International Hellenic University, qualitative/mixed methods, chronic care.
4. Gender	Was the researcher male or female?	CK: Male; TB: Female.
5. Experience and training	What experience/training did the researcher have?	CK had training in qualitative methods and clinical experience in chronic care; TB had extensive teaching/research experience in qualitative and mixed methodologies.
6. Relationship established	Was a relationship established prior to study commencement?	No prior professional or personal relationship with participants.
7. Participant knowledge of interviewer	What did participants know about the researcher?	They were informed verbally and in writing about researcher's role, aims of study, and affiliation.
8. Interviewer characteristics	What characteristics were reported about the interviewer/facilitator?	Reflexive notes taken; bracketing strategies used; regular discussions with senior

		researcher (TB) to minimize bias.
**Domain 2: Study design**		
9. Methodological orientation and theory	What methodological orientation underpinned the study?	Thematic analysis (Braun & Clarke, 2006), inductive approach.
10. Sampling	How were participants selected?	Purposive sampling through "Help at Home" program staff.
11. Method of approach	How were participants approached?	Via "Help at Home" staff, then contacted by PI with verbal and written information.
12. Sample size	How many participants?	17 total (8 patients, 9 caregivers).
13. Non-participation	How many people refused and reasons?	15 patients & 16 caregivers approached; 5 declined (poor prognosis), 4 declined (time/interest), 5 declined (logistics/family constraints).
14. Setting of data collection	Where was data collected?	Participants' homes.
15. Presence of non-participants	Anyone else present during interviews?	Only participant and interviewer (unless caregiver was also part of sample).
16. Description of sample	What are the sample characteristics?	Table 2 shows demographics, conditions, caregiver relationships, and income.
17. Interview guide	Were questions, prompts, guides provided by authors?	Yes, semi-structured guides co-developed by team, iteratively refined.
18. Repeat interviews	Were repeat interviews carried out?	No repeat interviews reported.
19. Audio/visual recording	Did the research use recording?	Yes, audio-recorded.
20. Field notes	Were field notes made?	Yes, immediately after each interview.
21. Duration	What was the duration of interviews?	40–85 minutes.
22. Data saturation	Was data saturation discussed?	Yes, reached when no new codes/themes emerged in last two interviews.
23. Transcripts returned	Were transcripts returned to participants for comment/correction?	No transcript return; reflexivity ensured authenticity.
**Domain 3: Analysis and findings**		
24. Number of data coders	How many coders?	Two (CK and TB).
25. Description of coding tree	Did authors provide a description?	Yes, sample codebook with 6–8 codes in Supplementary Table S1.
26. Derivation of themes	Were themes identified in advance or derived from data?	Inductively derived, no predetermined framework.
27. Software	What software was used?	NVivo 14.
28. Participant checking	Did participants provide feedback on findings?	No direct member-checking; triangulation and reflexive dialogue used.
29. Quotations presented	Were participant quotations presented?	Yes, Boxes 1–5 contain illustrative quotations with identifiers (P-/C-).

30. Data and findings consistent	Was there consistency between data and findings?	Yes, results reflect direct quotations and analytic interpretations.
31. Clarity of major themes	Were major themes clearly presented?	Yes, five overarching themes described with subthemes and exemplar quotes.
32. Clarity of minor themes	Is there description of diverse cases or minor themes?	Yes, contradictory/outlier responses noted in Results.

Table S3. CASP Qualitative Checklist.

CASP Question	Assessment
1. Was there a clear statement of the aims of the research?	Yes. The study aimed to explore the palliative care needs of patients with advanced non-malignant chronic conditions to inform future patient-centred needs assessment tools.
2. Is a qualitative methodology appropriate?	Yes. A qualitative approach was suitable for exploring complex experiences and needs.
3. Was the research design appropriate to address the aims?	Yes. Semi-structured interviews enabled in-depth exploration of participants' experiences.
4. Was the recruitment strategy appropriate?	Yes. Purposive sampling with maximum variation ensured inclusion of information-rich cases.
5. Was the data collected in a way that addressed the research issue?	Yes. Interviews conducted in familiar settings facilitated open discussion.
6. Has the relationship between researcher and participants been adequately considered?	Yes. Reflexivity was maintained and no prior relationships existed.
7. Have ethical issues been taken into consideration?	Yes. Ethical approval was obtained, informed consent secured, and confidentiality ensured.
8. Was the data analysis sufficiently rigorous?	Yes. An iterative analytic process with independent coding and team discussions was applied.
9. Is there a clear statement of findings?	Yes. Findings are clearly presented and supported by participant quotations.
10. How valuable is the research?	The study provides valuable insights into patient needs in community-based palliative care and offers a strong qualitative foundation for future research and tool development.

Table S4. Excerpt of codebook and theme development.

Code	Operational Definition	Illustrative Quotation	Theme
**Dependence in daily activities**	Need for help with mobility, hygiene, eating, or other basic activities.	"I cannot bathe myself anymore; I need my wife for almost everything." (Patient 4)	Basic daily care and physical support
**Home-based medical needs**	References to need for regular visits from nurses, physiotherapists, or doctors.	"If the nurse could come once a week, I would feel safer." (Patient 1)	Basic daily care and physical support
**Emotional exhaustion of caregivers**	Descriptions of psychological fatigue, stress, or burnout from caregiving duties.	"I sometimes feel I am losing myself while taking care of him." (Caregiver 2)	Psychosomatic and emotional impact

**Fear and uncertainty about illness**	Expressions of anxiety, fear of deterioration, or uncertainty about the future.	"Every day I wonder what will happen next; it keeps me awake at night." (Patient 3)	Psychosomatic and emotional impact
**Loss of social roles**	Mention of reduced community participation or abandonment of hobbies and roles.	"I had to quit my choir… now my life is only about care." (Caregiver 5)	Social withdrawal and role change
Resilience / coping resources (family/spirituality/community)	References to coping resources and strengths used to endure and adapt to illness/caregiving at home (e.g., family support, faith/spiritual practices, community ties). This code emerged inductively as such narratives recurred across interviews.	"If I didn't have my husband, I would be lost." (Patient 2) / "Whatever happens, we don't give up. We pray and we keep going." (Caregiver 1)	Family, spiritual, and community resilience
**Financial constraints**	Difficulties covering medical expenses, home modifications, or private services.	"We can't afford a physiotherapist, so we just manage on our own." (Caregiver 6)	Barriers and unmet needs in organized care
**Need for professional psychosocial support**	Requests for psychological counseling, caregiver education, or structured support groups.	"We are not trained… we need guidance and someone to talk to." (Caregiver 4)	Barriers and unmet needs in organized care

Notes on coding and theme development

- **Transcription & anonymisation:** Interviews were transcribed verbatim and anonymised prior to analysis.
- **Familiarization with the data:** Transcripts were read repeatedly, with concurrent note-taking to capture initial analytic impressions.
- **Generating initial codes (coding data extracts):** Data extracts were coded line-by-line, generating initial codes (labels applied to meaningful segments of text), using participants' own words where possible. Two independent coders reviewed each transcript.
- **Collating/grouping related codes:** Related codes were collated and grouped to support pattern identification across transcripts (without introducing intermediate "categories").
- **Generating candidate themes:** Grouped codes were synthesized into candidate themes that captured shared patterns of meaning relevant to the research aims.
- **Reviewing/refining themes:** Candidate themes were reviewed and refined iteratively—first at the level of coded extracts and then across the entire dataset—to ensure internal coherence and distinctiveness between themes.
- **Defining and naming final themes:** Final themes were defined and named, resulting in five themes as presented in the Results.
- **(Optional) Developing subthemes:** Where needed, subthemes were developed to structure and break down broader themes and improve clarity of presentation.
- **Producing the report/write-up:** The final report integrated an analytic narrative with illustrative quotations supporting each theme.

- Both semantic codes (explicit statements) and latent codes (underlying meanings, such as hidden caregiver burden) were retained.
- **Codebook transparency:** A fuller version of the codebook with additional codes and quotations is available upon request.

Table S5. Individual Demographic and Clinical Profiles of Patients and Their Primary Informal Caregivers (Pseudonyms).

PATIENT ID	Gender	Age	Chronic condition(s)	Duration of Illness*	CAREGIVER ID	Gender	Age	Relationship to Patient**
P-01	Female	92	Heart Failure; Chronic Kidney Disease	Not reported in interview	C-01	Female	*82*	*Spouse*
P-02	Female	86	Dementia; Diabetes Mellitus; Parkinson's disease	Not reported in interview	C-02	Male	*67*	*Son*
P-03	Male	81	Heart Failure; Chronic Respiratory Disease	Not reported in interview	C-03	Female	*60*	*Daughter*
P-04	Female	86	Parkinson's disease; Diabetes Mellitus; Hypothyroidism	Not reported in interview	C-04	Male	*64*	*Son*
P-05	Female	89	Heart Failure	Not reported in interview	C-05	Male	*83*	*Spouse*
P-06	Female	53	Multiple Sclerosis	≈20 years (explicitly reported; long-term wheelchair use)	C-06	Female	*57*	*Other family member (daughter-in-law)*
P-07	Female	88	Rheumatoid Arthritis; Chronic Pain	Long-standing condition (exact duration not specified)	C-07	Female	*49*	*Other family member (daughter-in-law)*
P-08	Female	90	Heart Failure; Diabetes Mellitus	Not reported in interview	C-08	Male	*79*	*Other family member (father)*
					C-09	Female	*73*	*Other family member (mother)*

P-xx denotes patients and C-xx denotes primary informal caregivers. All identifiers are pseudonyms used to ensure participant anonymity. The order of patients and caregivers in the table is for presentation purposes only and does not imply a one-to-one correspondence between Patient ID and Caregiver ID. This approach was adopted to prevent any potential identification of participants or inference of relationships between individuals.Patients may present with more than one chronic condition; therefore, disease categories are not mutually exclusive.

*The duration of illness was not systematically collected, as the interviews focused on participants' current experiences and palliative care needs rather than detailed clinical histories. In addition, several participants were unable to recall precisely when their illness began or how long they had been living with their condition, reflecting the long-standing and progressive nature of their diseases. For these reasons, duration of illness was not used as an analytical variable in the present study.

** Caregiver relationship categories were grouped for analytical purposes; "other family members" include in-laws and parents caring for adult children.
